# Czerny’s Operation for the Radical Cure of Hernia

**Published:** 1880-04

**Authors:** 


### CZERNY'S OPERATION FOR THE RADICAL CURE OF HERNIA.

Of the many operations advocated for the radical cure of hernia, none have been proposed, thus far, that are free from objection of one kind or another. The advantages claimed by Czerny for the operation adopted by him are, that the results are more certain, as the fibrous columns of the external ring are drawn close together, and an exact application of the sutures can be made, causing a narrowing of the internal aperture. The operation is as follows: An incision is made along the whole length of the hernia. If intestinal and reducible, the contents of the sac are returned to the cavity; if irreducible and intestinal, they are dissected loose and reduced, the sac being ligatured at the neck and cut off. When the contents are omental, they are included in the ligature with the sac. The fibrous columns of the external ring are drawn together by an interrupted suture of strong carbolized catgut. After the insertion of a drainage-tube, the wound is closed with sutures and dressed antiseptically. This mode of procedure has been adopted in nine cases of inguinal hernia, without the occurrence of peritonitis.—*Chicago Medical Gazette, Jan., 1880.*

